# Breaking Barriers: CDC and American Diabetes Association Unite to Combat Diabetes

**DOI:** 10.5888/pcd22.240273

**Published:** 2025-02-27

**Authors:** Christopher S. Holliday, Robert A. Gabbay

**Affiliations:** 1 Centers for Disease Control and Prevention, Division of Diabetes Translation, Atlanta, Georgia; 2 American Diabetes Association, Alexandria, Virginia

Diabetes is one of the most serious health problems our country has ever faced, ranking as the eighth leading cause of death in the US (1). More than 38 million adults have diabetes, and 98 million have prediabetes (2). Medical costs for people with diabetes are double the costs for those without — in fact, diabetes is the most expensive chronic condition, with health care costs and lost work and wages totaling $413 billion a year (2). Differences in the occurrence of diabetes and its related complications depend on factors such as income, geographic location, education level, race, and ethnicity (1). To tackle these disparities, it may be essential to have a clear understanding of the social determinants of health (SDOH) that lead to them. SDOH are nonmedical factors that significantly influence health outcomes (3), including access to quality health care, stable housing conditions, safe neighborhoods, built environment features that facilitate healthy eating and physical activity, and economic stability factors such as employment and educational opportunities (4).

The toll of diabetes on our country is significant (1,2). Combatting the effects may require a comprehensive, multilayered approach encompassing leadership, research, prevention, management programs, and policies at all levels of the socio-ecological strata — individual, interpersonal, community, and society. The Centers for Disease Control and Prevention (CDC) has collaborated with the American Diabetes Association (ADA) and other national partners, federal agencies, state and local health departments, health care providers, and community organizations to address the devastating impact diabetes has on the nation (2).

For nearly 50 years, CDC’s Division of Diabetes Translation (DDT) has been at the forefront of the fight against diabetes (5). To begin reversing the epidemic, CDC aims at a 1% reduction in incidence per year by the end of 2030 (6). CDC provides funding to support individual-level efforts to prevent or delay the development of type 2 diabetes among at-risk individuals and population-wide approaches to address SDOH. CDC also tracks progress toward meeting established national goals and objectives (including Healthy People 2030) and informs policy and program development.

ADA aims to stem the rise in new cases of diabetes while ensuring a reduction of diabetes complications (7). Each year, ADA identifies research priorities for investment that align with these overall goals. The yearly publication of the *ADA Standards of Care* provides clinical guidelines for preventing and treating diabetes (7). These standards are the foundation for efforts to aid professionals and people with diabetes in moving from knowledge to action. They include recommendations that clinicians should assess for SDOH or nonmedical health-related needs to inform treatment decisions. This is augmented by continuing education courses in the ADA Institute of Learning, which provides training programs for diverse learners. Guidelines are then disseminated and implemented through quality improvement projects and science-based interventions (7,8).

This commentary discusses efforts by CDC and ADA to address the upstream factors that exacerbate the incidence and complication rates of diabetes. Specific focus is on the “upstream,” “midstream,” and “downstream” approaches that are being taken to tackle this issue (Table) (38). Attention is also given to the potential areas of opportunity that could be leveraged to enhance public health strategies aimed at mitigating the health and economic consequences of diabetes.

**Table:** Upstream, Midstream, and Downstream Approaches the Centers for Disease Control and Prevention’s (CDC’s) Division of Diabetes Translation (DDT) and the American Diabetes Association (ADA) Are Leading to Address the Socio-Contextual Factors That Exacerbate the Incidence and Complication Rates of Diabetes

Approach and organization	Activities
**Upstream — population-level activities addressing social determinants of health (SDOH)**	
**CDC/DDT**	• Increase access to food programs as part of the National Diabetes Prevention Program (National DPP) lifestyle change program (9). • Enhance access to community–clinical linkage programs by developing multidirectional e-referral systems that support the electronic exchange of information between health care and community-based organizations (10). • Improve the capacity of the diabetes workforce to address factors related to SDOH that affect outcomes for priority populations with and at risk for diabetes (10). • Implement through the Simulation Model of Interventions Linking Evidence to SDOH (SMILES) Simulation Project various interventions, policies, and strategies that effectively reduce inequities in chronic outcomes (11). • Develop a portfolio of evidence-based change strategies through a knowledge to practice project aimed to address policy and systems-level approaches, including early diagnosis, immediate linkage to care, retention in care, and improved clinical outcomes.^a^
**ADA**	• Advocate for access to healthy food (12). • Expand accessible treatments and technology (13). • Train and expand use of community health workers to help address SDOH barriers (14,15). • Fund research and implement health equity initiatives (16). • Empower communities and people living with diabetes with information on healthy lifestyles (17). • Scale innovative community engagements (18).
**Midstream — individual-level type 2 diabetes prevention activities**	
**CDC/DDT**	• Raise awareness of prediabetes and risk for type 2 diabetes through the nationwide campaign, “Do I Have Prediabetes?” The campaign aims to help people take steps to prevent or delay type 2 diabetes by taking the 1-minute prediabetes risk test and knowing their risk (19). • Increase enrollment and retention of priority populations in the National DPP and Medicare Diabetes Prevention Program lifestyle interventions (10). • Work with partners to expand access to the National DPP lifestyle intervention as a covered health benefit (10). • Build capacity for National DPP program suppliers to bill via umbrella hub arrangements. • Address childhood obesity and diabetes risk reduction by expanding access to family healthy weight programs (10). • Develop an open research agenda through the Lifestyle Change Implementation Research Network to improve the equity of enrollment and retention in lifestyle change interventions.^a^
**ADA**	• Expand diagnosis and treatment of obesity and prediabetes (18). • Work to improve access to diabetes anti-obesity medications (13). • Fund research to identify long-term approaches to healthier lifestyles (20).
**Downstream — management and care for people with diabetes; prevention of diabetes-related complications**	
**CDC/DDT**	• Fund programs to increase access to nutritious food for people with, or at risk for, diabetes, heart disease, obesity, cancer, and kidney failure (eg, Food is Medicine) (9,21). • Strengthen self-care practices by improving access and participation in diabetes self-management education and support (DSMES) services (10). • Develop an integrated DSMES data system to support coordination of programs across the country.^a^ • Prevent diabetes complications for priority populations through early detection of chronic kidney disease and diabetic retinopathy (10). • Improve quality of care for priority populations with diabetes, including Increasing adoption or enhancement of team-based care for people with diabetes supported by sustainable payment models (22–24). Increasing adoption and use of clinical systems and care practices (eg, health information technology and electronic health records, clinical decision-support tools, learning collaboratives) (25–28). • Build and strengthen a sustainable infrastructure for community health workers to expand their involvement in evidence-based diabetes management programs and services (10,15). • Conduct surveillance and publish CDC’s National Diabetes Statistics Report (1). • Support health-related social needs screening in prevention and management (10). • Increase participation of Black or African American persons with diabetes in DSMES programs through demonstration projects such as Communities United Together to Manage Diabetes (CUT2MD) and Communities United Together for Health (CUT4Health), which integrate barbers and stylists as trusted community members in interventions (29,30).
**ADA**	• Provide authoritative guidance on therapeutic diabetes education worldwide through the diabetes Education Recognition Programs (ERP), ensuring broad adoption of the standards of DSMES services (31). • Provide support to over 3,700 sites, serving around 800,000 people with diabetes per year with the latest innovations delivered via ERP (31). • Provide support to ensure the latest innovations were adopted (31,32). • Increase access to DSMES in areas with high prevalence of diabetes (31,33). • Increase referrals to DSMES by providers and expand services in primary care (34,35). • Expand the availability of the ADA-recognized DSMES services as a covered health benefit for Medicaid beneficiaries (34,35). • Use DSMES data to develop training and technical assistance to improve outcomes.^a^ • Increase access to continuous glucose monitoring for those at highest risk for diabetes-related complications (32). • Develop and implement a health professional education campaign (through the ADA Institute for Learning) to screen, treat, and refer patients with diabetes for diabetes-related complications and for SDOH needs (36). • Publish the annual Standards of Care and the Abridged Standards of Care, which target primary care professionals, to be more interactive and easily digestible for busy health care professionals (7). • Fund translational research that emphasizes interventions to reduce inequities (20). • Publish latest research on addressing health disparities (20). • Advocate for federal law that gives students the right to receive the diabetes care they need to be safe and participate in school activities just like any other child. Help families receive equal access to care through the Safe at School program (37). • Engage community health workers through the establishment of learning opportunities in diabetes and prediabetes through the ADA Institute of Learning and in community settings. Highlight the value of community health workers in the Standards of Care, to provide the evidence base that is leading to reimbursement for their services (14,15,36).

a These activities are in the early stages of program development. Public access is forthcoming.

## Upstream Socio-Contextual Factors and Approaches

Where a person lives, works, learns, worships, and plays can significantly affect their health (39). The upstream SDOH influencing diabetes incidence and prevalence impact chronic disease overall: access to nutritious, affordable foods (40,41), opportunities to exercise and live in safe environments (42), access to quality health care (43,44), and structures and policies that support equity (38). For example, type 2 diabetes prevention and management are challenging when people must travel long distances to see a health care professional (45); cannot afford medications and technology, such as continuous glucose monitoring devices and automatized insulin delivery (16,17); or have limited access to healthy food (46,47) and safe places to engage in physical activity (17,48,49).

Congress charged the National Clinical Care Commission (NCCC) to make recommendations to leverage federal policies and programs to improve diabetes outcomes (50). A fundamental recommendation is that health equity should be integrated into all federal policies and programs that affect people at risk for or with diabetes (51). Additional recommendations focus on lifestyle change programs and medications that have the greatest likelihood of preventing diabetes in those who are at high risk for type 2 diabetes, specifically people with prediabetes, and on access to, participation in, and sustainability of these interventions (52). Simultaneously, to address the disparity in available resources for people with diabetes, recommendations include providing insurance coverage for high-value treatments and creating a quality measure that improves patient safety by reducing the intensity of treatment of high-risk patients (50).

Policies, systems, and environmental change strategies can substantially affect health outcomes at a population level (53,54). When implemented with a health equity lens, the effectiveness of these approaches can be transformative for all populations. These upstream interventions tend to be complex to institute and often take significant time and resources (17). CDC and ADA both lead programs and initiatives that deploy upstream strategies to increase access to nutritious foods, access to quality health care, services for diabetes prevention and management, and opportunities to exercise in safe environments (Table). The efforts outlined in the table are in largely formative and early implementation stages. These efforts are closely aligned with core CDC and ADA missions, which in turn contribute to fulfilling the reporting requirements stipulated by the Government Performance and Results Act (55).

A systematic approach was used to identify the selected initiatives. CDC/DDT conducted comprehensive external landscape and internal factors analyses to identify forces influencing diabetes management and prevention and develop a 5-year strategic plan (56). Key performance indicators play a crucial role in tracking progress toward goals and intended outcomes, ensuring accountability and effectiveness (55). Programs and initiatives, such as the partnership between CDC/DDT and ADA aimed at enhancing access to community–clinical linkage programs, incorporate capacity-building and evaluation components supported by established performance measures that meet CDC/DDT grant deliverables (57–59). ADA also evaluates each specific ADA program. The established evaluation plan includes short-, intermediate-, and long-term evaluation components that allow teams to scale high-impact efforts based on lessons learned. Qualitative and quantitative metrics align with key inputs and are collaboratively designed to measure progress toward long-term goals. An evaluation team meets every 2 weeks to monitor performance for continuous improvement.

## Midstream Approaches to Individual-Level Type 2 Diabetes Prevention

Midstream interventions — geared toward people at high risk for type 2 diabetes — could also be advanced. Primary prevention of type 2 diabetes begins with awareness and continues through behavior change among those at risk. CDC and ADA worked with the Ad Council in 2016 to launch a national campaign to raise awareness of prediabetes (52). CDC continues to lead the *Do I Have Prediabetes?* campaign to date. Research shows that once a person is aware that they have prediabetes, they are more likely to make healthy lifestyle changes to prevent or delay type 2 diabetes (52).

CDC’s National Diabetes Prevention Program (National DPP) is a partnership of public and private organizations working to build a nationwide delivery system for a lifestyle change program proven to prevent or delay onset of type 2 diabetes in adults at high risk. CDC aims to increase the availability of quality programs, uptake from at-risk populations, referrals from health care providers, and coverage by public and private payers as well as by employers. Since April 2018, the National DPP lifestyle change program has been a covered preventive service for eligible Medicare beneficiaries under the Medicare Diabetes Prevention Program (MDPP). ADA, in collaboration with CDC, has been actively working on enhancing the infrastructure to support the widespread expansion of the National DPP and MDPP.

## Downstream Approaches to Diabetes Management and Prevention of Diabetes-Related Complications

Varied downstream approaches may also be necessary to improve the care of people with diabetes and to prevent and manage complications. Diabetes is frequently associated with macrovascular (eg, heart disease, stroke) and microvascular (eg, retinopathy, nephropathy, neuropathy) complications, lower-extremity amputations, and acute events such as diabetic ketoacidosis. Despite new treatments and technologies for people with diabetes, many of these outcomes have not substantially improved (eg, lower-limb amputation rates). For example, between 2000 and 2018, the percentage of diabetes-related hospitalizations out of all hospitalizations in the US increased from 17.1% to 27.3% (60). Hospitalizations are more prevalent among certain demographic groups (eg, low income), suggesting the differential distribution of diabetes-related complications or inequalities in health care access and usage. As patients with diabetes live longer, the burden of complications and consequent hospitalization rates are expected to increase (2).

Both CDC and ADA are leading multilevel initiatives to address health care and educational needs of individuals with diabetes to better manage their disease and prevent complications. Through diabetes self-management education and support (DSMES) services, people with diabetes learn and develop new skills in monitoring blood glucose, healthy eating, physical activity, coping, medication adherence, risk reduction, and problem-solving. While DSMES services are effective in preventing or delaying diabetes complications, fewer than 7% of people participate within the first year of their diabetes diagnosis (61). CDC and ADA (a national accrediting organization for DSMES) are working with partners and providers to increase the referrals to these programs and help people access culturally and linguistically appropriate services.

## Looking to the Future

Our organizations are united in the long-term goal of seeing a world free from the devastation of diabetes. However, that can only happen when we work together across all sectors to eliminate disparities and address obstacles that keep people from taking those critical first steps to prevent or manage their diabetes. To meaningfully address the complexities at all levels — upstream, midstream, and downstream — strategies that include policy, systems, and environmental change can be essential. Such strategies may include the following:

- Evidence-based interventions that support the prevention of type 2 diabetes and help people with diabetes to live well.
- Activities such as 1) expanding the role of pharmacies and team-based care approaches, including less traditional providers (eg, pharmacists, dentists and dental hygienists, behavioral health and community health workers [CHWs]) as part of the team in delivery of DSMES and the National DPP lifestyle intervention program; 2) innovative service delivery and payment models; 3) infrastructure to support these providers; 4) two-way information exchange between health care and community-based organizations; and 5) partnerships with AARP and Centers for Medicare and Medicaid Services to increase enrollment and retention for the MDPP.
- Environmental change strategies that ensure that all people in the US, regardless of life circumstances, have access to healthy food and the ability to be physically active.

We recognize that significant challenges face us as we forge ahead with these strategies. However, we remain optimistic. The evidence shows that there are strategies that can successfully address these challenges (17). We are committed to bringing together partners from governmental and private sectors, public health, and clinical medicine, along with engaged individuals, in a united effort to reduce the risk for type 2 diabetes and to provide those living with diabetes the support they need to manage the disease and live well. Through public health leadership, partnership, research, programs, and policies that translate science into practice, CDC and the ADA are united in achieving our mission to reduce the preventable burden of type 2 diabetes in the US.
